# Diagnóstico ecográfico precoz de enfermedad de Crohn

**DOI:** 10.1016/j.aprim.2024.103087

**Published:** 2024-09-10

**Authors:** Luis Ortiz-González, Carlos Ortiz-Peces, Luis Ortiz-Peces

**Affiliations:** a Departamento de Ciencias Biomédicas, Facultad de Medicina y Ciencias de la Salud, Universidad de Extremadura, Badajoz, España; b Clínica de Pediatría Dr. Luis Ortiz, Badajoz, España; c Servicio de Cirugía Oral y Maxilofacial, Hospital Universitario La Paz, Madrid, España

En la actualidad, seguimos necesitando un consenso para determinar la mejor técnica de imagen diagnóstica de la enfermedad inflamatoria intestinal (EII) en la población pediátrica y, a menudo, no está claro qué modalidad elegir en circunstancias clínicas específicas^1^.

La enfermedad de Crohn (EC) pediátrica suele ser más grave, requiere niveles más altos de inmunosupresión y se asocia con una mayor morbilidad en comparación con la EC en adultos. Las opciones de tratamiento son también limitadas y se requiere el uso de terapias no autorizadas^2^.

El diagnóstico precoz de EII es crucial en el niño. El 25% de los niños con EC presentan síntomas durante más de un año antes del diagnóstico debido a la sintomatología inespecífica^3^.

El retraso en el diagnóstico puede provocar efectos adversos, como el deterioro del crecimiento, el retraso puberal, la enfermedad ósea, la longevidad de la carga de enfermedad y el impacto psicosocial^2^.

Los niños con EII están expuestos a radiaciones ionizantes como consecuencia de múltiples estudios de imagen realizados en el diagnóstico inicial, durante el tratamiento, el período de seguimiento y la valoración de posibles complicaciones que conviene plantearse en el diagnóstico diferencial de multitud de procesos intercurrentes que acontecen a lo largo de su vida^1^.

La ecografía abdominal, por su bajo costo, no invasividad y amplia disponibilidad, ha surgido como una modalidad de imagen alternativa para el diagnóstico y seguimiento de la EC en niños, y algunos estudios, principalmente en adultos, muestran una sensibilidad y especificidad comparables a la enterografía por tomografía computarizada y resonancia magnética (RM)^4^.

La ultrasonografía tiene la capacidad de detectar, localizar y caracterizar con precisión la inflamación de la pared intestinal y evaluar las anomalías adyacentes, con un buen valor predictivo negativo para la EII, mayor para la EC que para la colitis ulcerosa. Los cambios patológicos del intestino inflamado se pueden dividir esencialmente en hallazgos murales y extramurales. Estos últimos afectan al mesenterio circundante, que aparece engrosado con alteración del tejido adiposo y ganglios linfáticos reactivos hiperplásicos.

Con la ecografía abdominal se pueden identificar cambios murales en la pared intestinal, que puede engrosarse y mostrar ecogenicidad alterada (hipo o hiperecogenicidad), pérdida de estratificación en sus estadios más avanzados, aumento de la señal Doppler color, que denota hiperemia, y disminución relativa o falta de peristaltismo, como marcador de rigidez^5^.

Se utiliza actualmente para el cribado en niños con sospecha de EII con buen valor predictivo negativo^6^. Se postulan diferentes valores de espesor de la pared como umbral para un diagnóstico positivo sugerente de EII (de 1,5 a 3 mm para el íleon terminal y < 2 mm para el colon)^5^.

Con la creciente incorporación de la ecografía a pie de cama *(point of care)* como una herramienta más de apoyo al diagnóstico clínico en las consultas de atención primaria, de medicina de familia y pediatría, y de urgencias, y el elevado número de pacientes que presentan semiología clínica abdominal inespecífica, como dolor abdominal y diarrea, resulta necesario familiarizarse con las características ecográficas de esta enfermedad^1^.

Presentamos el caso de un niño de 9 años de edad, sin antecedentes personales y familiares de interés clínico, que consulta por presentar deposiciones líquidas y molestias abdominales inespecíficas de 2 semanas de evolución. En la exploración física presenta discreto grado de dolor abdominal difuso a la palpación profunda del mismo sin signos de irritación peritoneal. Se realizó una ecografía clínica abdominal que puso de manifiesto un peristaltismo disminuido y un engrosamiento mural del íleon terminal, con estratificación mural aún conservada, sugerente de EC en las fases iniciales de la enfermedad, que se confirmó mediante RM en la unidad de gastroenterología pediátrica (Figura 1, Figura 2).

**Figura 1:**
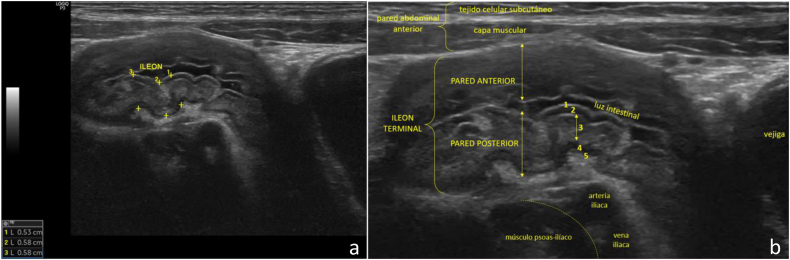
a) Corte transversal con sonda lineal de alta frecuencia (8-12 MHz) a nivel de fosa iliaca derecha donde se objetiva una sección longitudinal del íleon terminal con engrosamiento mural generalizado y cuantificación del espesor de su pared posterior (varias mediciones). b) Corte similar al previo donde se pone de manifiesto la conservación de la estratificación normal de la pared del íleon terminal en fases iniciales de la enfermedad, con engrosamiento de la misma a expensas de la submucosa, que se identifica hiperecogénica. Se ilustran 5 capas concéntricas alternativamente hiperecogénicas e hipoecogénicas: 1, interfaz mucosa superficial; 2, mucosa profunda, incluida la muscularis mucosa; 3, submucosa; 4, muscular propia; 5, serosa.

**Figura 2:**
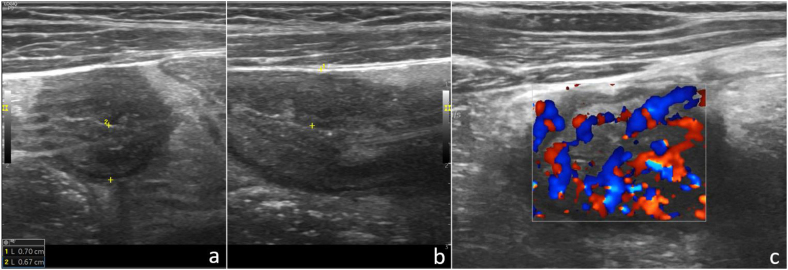
Corte longitudinal (a) y transversal (b) con sonda lineal de alta frecuencia (8-12 MHz) a nivel de fosa iliaca derecha donde se identifica el íleon terminal con engrosamiento generalizado de su pared y cuantificación de su espesor. c) Imagen similar a la primera donde se evidencia un aumento de la vascularización del íleon mediante Doppler color.

## Financiación

Los autores manifiestan que no han recibido financiación alguna para la elaboración del manuscrito.

## Consideraciones éticas

Los autores confirman que se han obtenido todos los consentimientos requeridos por la legislación vigente para la publicación de cualquier dato personal o imágenes de pacientes, sujetos de investigación u otras personas que aparecen en los materiales enviados a Elsevier, se han realizado todos los procedimientos éticos y se han respetado los derechos de privacidad de los sujetos humanos.

Los autores conservan una copia escrita de todos los consentimientos y, en caso de que Elsevier lo solicite, aceptan proporcionar las copias o pruebas de que dichos consentimientos han sido obtenidos.

## Declaraciones de interés

Ninguna.
